# Comparison of the Efficacy of Light’s Criteria With Serum-Effusion Albumin Gradient and Pleural Effusion Glucose

**DOI:** 10.7759/cureus.43319

**Published:** 2023-08-11

**Authors:** Karan Sharma, Lekhini Fultariya, Priya Reddy Mallimala, Kavita Shah, Vishal Sharma

**Affiliations:** 1 Internal Medicine, Poonam Multispeciality Hospital, Ahmedabad, IND; 2 Internal Medicine, Gujarat Cancer Society (GCS) Medical College, Hospital and Research Centre, Ahmedabad, IND; 3 Medicine, Kurnool Medical College, Bangalore, IND; 4 Medicine, University of North Carolina Medical Center, Chapel Hill, USA

**Keywords:** pleural effusion, transudate, exudate, glucose, seag, light’s criteria

## Abstract

Introduction

While Light’s criteria exhibit high sensitivity (98%) in detecting exudative pleural effusions, the capacity to rule out transudates is relatively limited. A previous study showed that approximately one-fifth of patients with congestive cardiac failure on diuretics also met the criteria for exudate. This study compares the diagnostic value of Light’s criteria, the serum-effusion albumin gradient (SEAG) method, and pleural effusion glucose levels for accurately categorizing pleural effusion as transudate or exudate.

Methodology

We conducted this cross-sectional observational study in a tertiary care hospital in Ahmedabad, India. Two hundred patients with pleural effusion undergoing thoracentesis were included. Laboratory parameters measured in pleural fluid analysis included pleural fluid protein, pleural fluid lactate dehydrogenase (LDH), pleural fluid albumin, and pleural fluid glucose. Serum protein, serum LDH, and serum albumin were also collected. Mean values and standard deviations (SDs) were calculated for analysis.

Results

A significant difference was observed in the mean value of exudative and transudative effusions for each parameter (pleural fluid protein/serum fluid protein ratio, pleural fluid LDH/serum fluid LDH ratio, pleural fluid LDH, SEAG, and pleural fluid glucose) (P < 0.001). Light’s criteria demonstrated the highest efficacy in diagnosing exudates (accuracy = 97.50%), while SEAG demonstrated the highest efficacy in diagnosing transudates (accuracy = 97.50%).

Conclusion

SEAG is an effective alternative diagnostic tool for identifying transudates misclassified by Light’s criteria. Its use can contribute to prompt diagnosis and timely treatment of patients with pleural effusion, improving patient outcomes.

## Introduction

Pleural effusion is the abnormal accumulation of fluid in the pleural cavity between the visceral and parietal pleurae [1]. The fluid that permeates into the pleural cavity through intact pulmonary vessels, e.g., in congestive heart failure (CHF), is called a transudate. Conversely, exudate is a fluid that escapes into the pleural cavity through lesions in blood and lymph vessels caused by inflammation. Transudative effusion is typically caused by a rise in hydrostatic pressure (congestive heart failure) and a fall in oncotic pressure (cirrhosis or nephrotic syndrome). By contrast, the causative factors for exudative effusions include infections (such as pneumonia or TB), malignancies (such as lung or breast cancer), pulmonary embolism, and pancreatitis [2].

Approximately one million people globally have pleural effusion every year [3]. Late diagnosis and treatment of pleural effusion can lead to severe complications and poor prognosis. Late diagnosis and untreated pleural effusions can lead to lung collapse, scarring, or sepsis, depending on the etiology. Differentiating between transudate and exudate plays a pivotal role in managing pleural effusion.

Light’s criteria are used to classify pleural effusion and state that in order to consider it an exudate, at least one of the following points must be satisfied: “pleural fluid protein/serum fluid protein ratio > 0.5, pleural fluid lactate dehydrogenase (LDH)/serum fluid LDH ratio > 0.6, or pleural fluid LDH > 2/3 the upper limit of normal serum LDH” [4].

The sensitivity of Light’s criteria in identifying exudative pleural effusions is high (98%); however, its ability to exclude transudates remains low. For instance, work by Porcel reported an almost 100% sensitivity for exudates but found that approximately one-fifth of patients with congestive cardiac failure on diuretics also met the criteria for exudate [5].

Parameters such as pleural fluid cholesterol, pleural fluid cholesterol/serum cholesterol ratio, alkaline phosphatase, pleural fluid/serum cholinesterase ratio, and pleural fluid/serum bilirubin concentration ratio can also be used to distinguish exudates from transudates [6-10]. None of the above tests have been able to surpass Light’s criteria in distinguishing exudative and transudative effusions because they often misclassify transudates as exudates in patients suffering from heart failure taking diuretics [4].

In order to avoid such misclassification, Light et al. proposed that serum-effusion albumin gradient (SEAG) be used when transudative pleural effusion is misclassified as exudative [11]. This study aimed to compare Light’s criteria with SEAG as well as pleural fluid glucose in differentiating transudative and exudative effusions. The latter method was included because determining pleural effusion glucose levels may also be critical in determining the nature of effusion since glucose levels < 60 mg/dL may suggest one or more of four things: parapneumonic effusion, malignant disease, rheumatoid disease, or tuberculous pleuritis [12].

## Materials and methods

After approval from the ethical committee, we conducted a prospective observational study at a tertiary care multispeciality hospital in Ahmedabad, India. Two hundred patients aged >14 years old suffering from pulmonary effusion undergoing thoracentesis in the pulmonology department were included. Sample collection was done from patients admitted to the inpatient medicine unit or intensive care unit. Written consent in English, Hindi, or Gujarati was taken from patients before their inclusion in the study. Patients aged <14 years, pregnant females, and all patients not giving consent were excluded.

The causative factor causing pleural effusion was determined by the following criteria: congestive heart failure was diagnosed on the basis of echocardiogram findings and the presence of lower extremity edema; nephrotic syndrome/hypoproteinemia was diagnosed on the basis of a urine dipstick showing ≥3+ proteins, 24-hour urine protein showing >3.5 g proteins/24 hours, and urine sediment microscopy showing fatty casts; the presence of malignant cells in either cytological examination or biopsy specimen confirmed the diagnosis of malignant pleural effusion; ascites, along with histopathological evidence, indicated the diagnosis of liver cirrhosis; and effusions caused by pneumonia were diagnosed on the basis of vital signs (temperature and oxygen saturation), microbiological examination of a sputum sample, and X-ray.

Tests performed on pleural fluid included total protein, LDH, albumin, and glucose estimations. Tests performed on serum included total protein, LDH, and albumin estimation. The following biochemical parameters were measured and computed. To calculate Light’s criteria, pleural fluid/protein ratio, pleural fluid/serum LDH ratio, and LDH concentration were measured. Serum-effusion albumin gradient (SEAG) was calculated by subtracting pleural effusion albumin concentration from serum albumin concentration. As suggested by previous studies, cutoff points were employed to differentiate transudates from exudates. For SEAG, a <1.2 g/dL cutoff value was used to classify as exudate, and for pleural fluid glucose levels, a <60 mg/dL cutoff value was used to classify exudates [13,14].

Blood and pleural fluid were centrifuged before total protein and LDH estimates on a fully automated system within 3-4 hours of sample collection. Total protein content was estimated by the Biuret method, and serum albumin by the bromocresol green (BCG) method. LDH was estimated by a modified International Federation of Clinical Chemistry (IFCC) method in which the rate of oxidation of nicotinamide adenine dinucleotide hydrogen (NADH) to nicotinamide adenine dinucleotide (NAD) was measured as a decrease in absorbance proportional to LDH activity. Albumin was measured by the BCG dye-binding method.

Statistical analysis was performed using Statistical Package for the Social Sciences (SPSS) version 21 (IBM SPSS Statistics, Armonk, NY, USA). Mean and range values were computed to determine the central tendency and spread of the data. T-test was used to compare the mean values of exudates and transudates while comparing the parameters of Light’s criteria, SEAG, and pleural fluid glucose. Sensitivity and accuracy analyses were calculated to compare the effectiveness of the two criteria. P-values < 0.05 were considered statistically significant.

## Results

**Figure 1:**
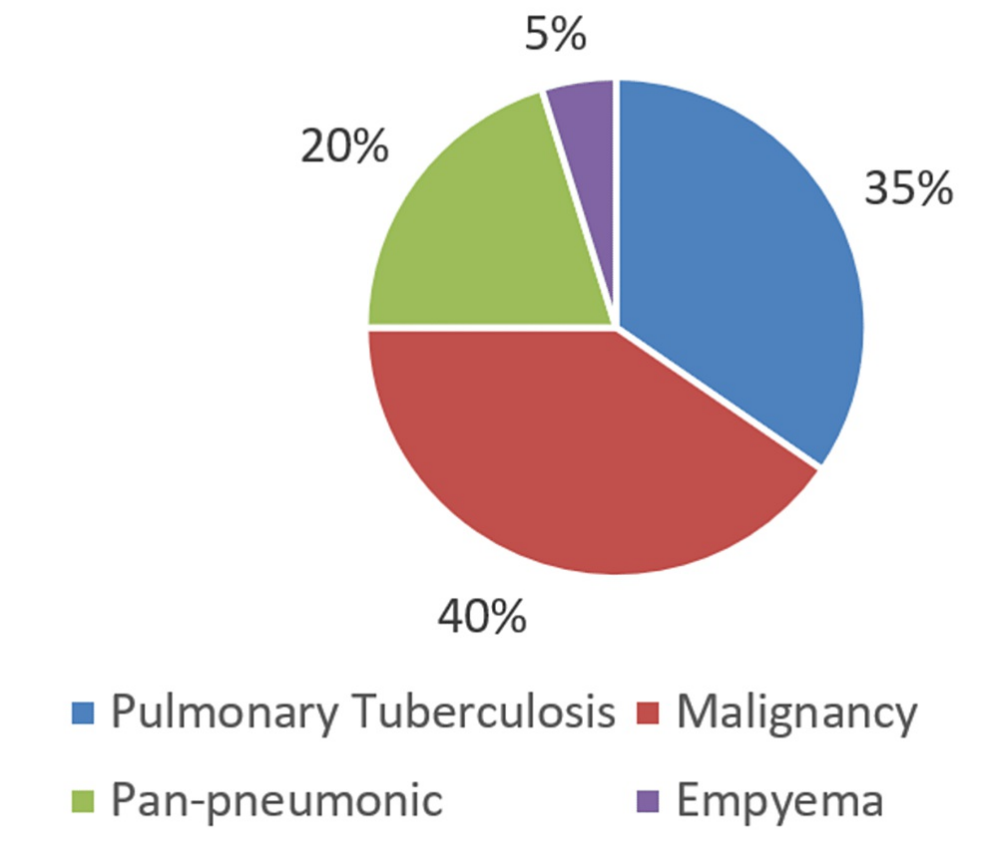
Number of patients with exudative pleural effusion (n = 104)

**Figure 2:**
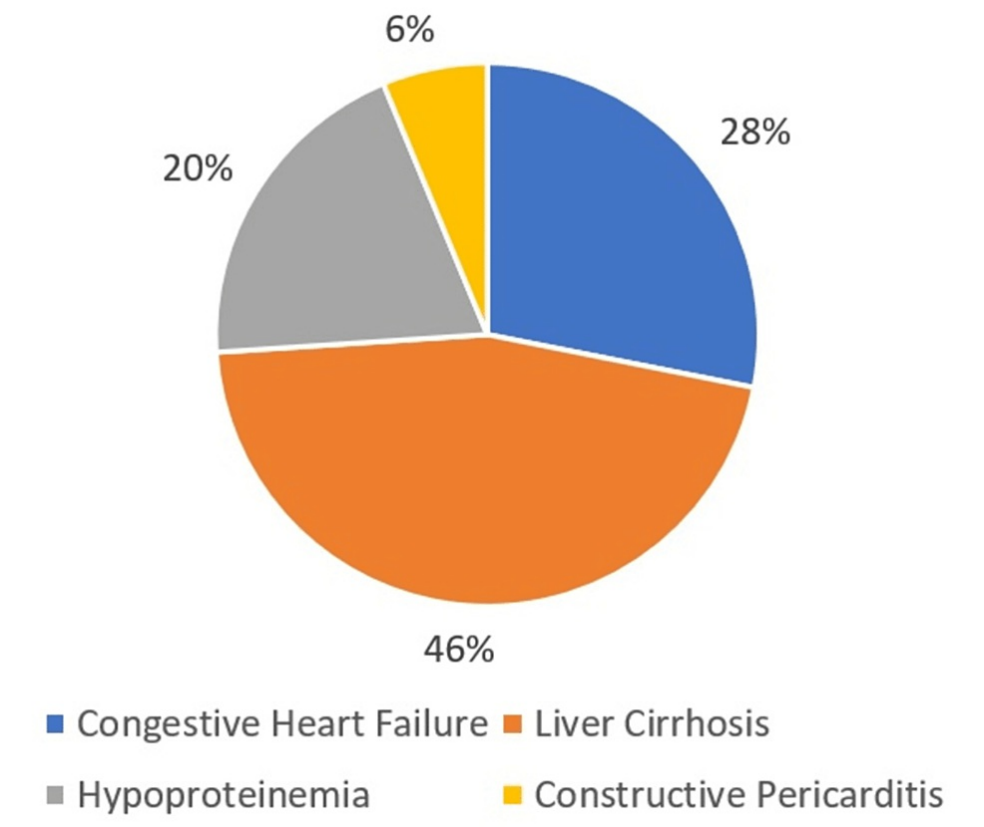
Number of patients with transudative pleural effusion (n = 96)

Based on our confirmatory gold standard test and the clinical and radiological diagnosis, the number of patients with exudative pleural effusion was 104 (Figure 1), while the number of patients with transudative pleural effusion was 96 (Figure 2).

Table 1 shows the distribution of exudative and transudative pleural effusions based on age. It was observed that the prevalence of transudative effusion increased with age, while young people had a higher incidence of exudative pleural effusion.

**Table 1:** Distribution of pleural effusion based on age

Age	Number of exudative effusions	Number of transudative effusions	Total
18-30	25	11	36
31-50	31	16	47
51-70	16	19	35
71-90	17	21	38
>91	15	29	44

Table 2 shows the distribution of exudative and transudative pleural effusion based on sex. Males were more often diagnosed with exudative pleural effusions than females, while females had more transudative pleural effusions. Table 3 shows the comparison of means of individual laboratory parameters. SEAG < 1.2 g/dL, LDH ratio > 0.6, protein ratio > 0.5, LDH levels > 2/3 of serum LDH, and pleural fluid glucose < 60 mg/dL were considered to be exudative pleural effusions. A significant difference was observed between the means of each parameter, as shown in Table 3 (P < 0.001).

**Table 2:** Distribution of pleural effusion based on gender

Gender	Number of exudative effusions	Number of transudative effusions	Total
Male	93	43	136
Female	11	53	64

**Table 3:** Comparison of means of different laboratory parameters used for the differentiation of transudative and exudative effusions SEAG: serum-effusion albumin gradient, LDH: lactate dehydrogenase

Laboratory parameter		Number of patients	Mean value	T-tests (independent)	P-value
SEAG	Exudates	104	0.79 ± 0.04	23.15	<0.001
	Transudates	96	1.59 ± 0.35		
Pleural fluid LDH/serum LDH	Exudates	104	0.5 ± 0.11	33.59	<0.001
	Transudates	96	0.11 ± 0.03		
Pleural fluid protein/serum protein	Exudates	104	0.76 ± 0	28.05	<0.001
	Transudates	96	0.43 ± 0.12		
Pleural fluid LDH	Exudates	104	1091.57 ± 540	11.84	<0.001
	Transudates	96	392.76 ± 214.35		
Pleural fluid glucose	Exudates	104	46.79 ± 11.5	9.38	<0.001
	Transudates	96	61.62 ± 10.8		

Table 4 shows the number of true-positive, false-positive, true-negative, and false-negative cases, which were used to calculate the various diagnostic evaluations such as sensitivity, specificity, positive predictive value (PPV), and accuracy of the three parameters.

**Table 4:** Data for calculating sensitivity, specificity, positive predictive value, and accuracy SEAG: serum-effusion albumin gradient

	Light’s criteria		SEAG		Pleural effusion glucose	
	Exudates	Transudates	Exudates	Transudates	Exudates	Transudates
True positive	102	86	96	94	94	84
False positive	3	6	4	3	14	12
True negative	93	98	92	101	82	92
False negative	2	10	8	2	10	12

Table 5 shows the sensitivity, specificity, positive predictive value (PPV), and accuracy of all three parameters. These data show that Light’s criteria have the highest accuracy when diagnosing exudative pleural effusions, while SEAG has the highest accuracy when diagnosing transudative pleural effusions.

**Table 5:** Sensitivity, specificity, positive predictive value, and accuracy of Light’s criteria compared to SEAG and pleural effusion glucose SEAG: serum-effusion albumin gradient

	Light’s criteria		SEAG		Pleural effusion glucose	
	Exudates	Transudates	Exudates	Transudates	Exudates	Transudates
Sensitivity	98.08%	89.58%	92.31%	97.92%	90.38%	87.50%
Specificity	96.88%	94.23%	95.83%	97.12%	85.42%	88.46%
Positive predictive value	97.14%	93.48%	96%	96.91%	87.04%	87.50%
Accuracy	97.50%	92%	94%	97.50%	88%	88%

## Discussion

It is crucial to diagnose the type of pleural effusion (exudative versus transudative) upon diagnosis using ultrasound-guided thoracentesis. Once the effusion type is confirmed, a differential diagnosis can be made. Kopcinovic et al. have suggested that exudative pleural effusion requires invasive procedures such as cytopathology, pleural biopsy, and thoracotomy, while transudative effusion requires no further testing [15].

Demographic characteristics

In this study, the highest number of patients with exudative effusions were in the middle-aged group (age 31-50), and the number of exudative effusions decreased with older age. This finding is similar to that previously reported by Sandeesha et al. [13]. Our study also showed that the proportion of transudative effusions increased with age, which aligns with a previous study conducted by Sahi and Dwivedi [16].

Comparison of means

The current study shows that the mean protein ratio, mean LDH ratio, and mean pleural fluid LDH are significantly higher in exudates compared to transudates, which agrees with Light’s criteria. Mean albumin gradients were significantly higher in transudates compared to exudates (P < 0.001). This finding is similar to that reported by Sandeesha et al. [13].

This study also showed a significant difference in means of pleural fluid glucose levels in transudative versus exudative fluids, with mean glucose levels of transudative fluids being higher. The lower glucose levels in exudative effusions are due to the metabolization of glucose by leukocytes and bacteria.

Evaluation of diagnostic parameters

This study demonstrates that Light’s criteria are superior (more accurate) in classifying exudates compared to SEAG and mean glucose level, while SEAG was superior (more accurate) in classifying transudates. This finding agrees with that of Sandeesha et al. [13].

Roth et al. conducted a study involving 59 patients to evaluate the classification of pleural effusions using SEAG. They utilized a cutoff value of 1.2 g/dL and found that all transudates and 39 out of 41 exudates were accurately classified. That study demonstrated that the SEAG method had a sensitivity of 87% and a specificity of 92% [17]. Our findings reported a higher sensitivity and specificity in reporting transudates compared to exudates, which are comparable to the above study.

The mean pleural fluid glucose had the lowest accuracy in diagnosing transudative and exudative pleural effusion, although the mean glucose level was significantly different in exudates and transudates. Vianna previously suggested that the lower the level of pleural effusion glucose, the more likely a diagnosis of parapneumonic effusion [18]. Approximately 15%-25% of patients with malignant pleural effusions have pleural fluid glucose levels < 60 mg/dL. In another study conducted by Rodriguez-Panadero and López Mejías, it was found that patients with a greater extent of tumor at thoracoscopy had glucose levels < 60 mg/dL [19].

Although this study gives a broader comparison in differentiating transudates and exudates, we could not measure the accuracy of Light’s criteria, SEAG, and pleural effusion glucose in diagnosing based on different etiological factors. Future studies should include rarer etiological factors such as drug-induced pleural transfusion and post-radiotherapy to get a more accurate idea.

## Conclusions

We recommend that, for routine purposes, Light’s criteria, SEAG, and pleural fluid glucose should all be assessed. Light’s criteria have more diagnostic utility than SEAG and pleural effusion glucose levels in diagnosing exudative pleural effusions. In contrast, SEAG had more diagnostic utility in diagnosing transudative ones. Pleural fluid glucose is an essential diagnostic indicator in determining the cause of pleural effusions, and severely low levels of pleural fluid glucose may indicate parapneumonic effusion or underlying malignancy. Assessing all three parameters will give a more holistic picture in diagnosing the type of effusion.
